# Experimental and Numerical Investigation of a Photoacoustic Resonator for Solid Samples: Towards a Non-Invasive Glucose Sensor

**DOI:** 10.3390/s19132889

**Published:** 2019-06-29

**Authors:** Said El-Busaidy, Bernd Baumann, Marcus Wolff, Lars Duggen, Henry Bruhns

**Affiliations:** 1 Department of Mechanical Engineering and Production, Hamburg University of Applied Sciences, 20099 Hamburg, Germany; 2 Mads Clausen Institute, University of Southern Denmark, 6400 Sønderborg, Denmark

**Keywords:** Photoacoustic spectroscopy, acoustic resonator, glucose

## Abstract

T-cell resonators have been used lately for non-invasive blood glucose measurements for photoacoustic spectroscopy on skin samples. A resonator has a significant role in determining the strength of the measured signal and the overall sensitivity of the sensor. Here we present results of the measurement of the photoacoustic signal of such a T-cell resonator. The signal is also modelled using the amplitude mode expansion method, which is based on eigenmode expansion and the introduction of losses in the form of loss factors. The measurement reproduced almost all the calculated resonances from the numerical models with fairly good agreement. The cause of the differences between the measured and the simulated resonances are explained. In addition, the amplitude mode expansion simulation model is established as a faster and computationally less demanding photoacoustic simulation alternative to the viscothermal model. The resonance frequencies from the two models differ by less than 1.8%. It is noted that the relative height of the amplitudes from the two models depends on the location of the antinodes within the different parts of the resonator. The amplitude mode expansion model provides a quick simulation tool for the optimization and design of macro resonators.

## 1. Introduction

The interest in photoacoustic spectroscopy (PAS) has increased with the development of new high power infrared laser sources [1]. The technique is highly sensitive, enabling the measurement of weakly absorbing and optically opaque samples that cannot be measured using transmission spectroscopy [2,3]. The photoacoustic (PA) effect has already demonstrated its potential for various applications with both gaseous and solid samples [3,4,5]. This has resulted in the proposal of numerous photoacoustic-based sensors. 

PA sensors typically use infrared laser radiation to excite the vibrational states of molecules. Thermal de-excitation leads to a local temperature elevation. Since the radiation is modulated, the heat released by the molecules is as well. This generates periodic pressure changes in the surrounding environment that are detected as acoustic waves using a microphone or a tuning fork [6,7,8].

The signal produced by the PA effect is, however, usually weak and needs to be amplified. This can be achieved by a modulation of the electromagnetic radiation at an acoustic eigenfrequency of the resonator, thus exciting the corresponding acoustic mode. This significantly boosts the PA signal and increases the detection sensitivity of the sensor. The amplification of the PA signal can be maximized by optimizing the shape of the resonator. In a purely experimental investigation, various resonator shapes must be tested since the optimal geometry for maximum signal amplification is not obvious. This would be extremely time consuming and expensive. Therefore, numerical simulation methods are preferred [9,10,11].

The viscothermal (VT) model is considered the most accurate numerical method for simulating PA signals [10]. It can accurately map the loss effects at the surfaces of the resonator, which are the dominant loss mechanisms in acoustic resonators. However, the VT model is computationally demanding, requiring a lot of memory space and simulation time. In this article, we investigate the amplitude mode expansion (AME) model which is faster and computationally less demanding [11] in a closed T-cell resonator. 

The T-cell resonators have lately been used in photoacoustic sensors for the non-invasive measurement of blood glucose levels [2,12]. One end of the resonator is left open to reduce the accumulation of humidity within the cell as well as air temperature and pressure fluctuations during measurements [2]. However, due to the complexity of accurately modelling the open end, we will, in a first step, restrict the investigation of the AME model to a closed cell in this article.

Glière et al. compared the results of the AME model to those of the VT model and a third approach in a micro-resonator by looking at a single resonance [10]. The comparison was carried out for a gaseous sample in a differential Helmholtz resonator. Baumann et al. applied the AME model to estimate the PA signal of nitric oxide molecules in an hourglass resonator [13]. They simulated a Gaussian shaped heat source across the resonator to mimic the absorption of the laser beam by the nitric oxide molecules across the resonator. Here, we will extend the study of the AME model by simulating the PA signal of a macroscopic T-cell over a wide frequency range from 8 kHz to 62 kHz. We are interested in the PA signal produced by a solid sample instead of a gaseous sample. The solid sample is placed at one of the openings of the resonator. The resonator is filled with air instead of a gaseous absorbing sample. PA signal measurement of the simulated resonator is carried out and compared to the simulated results.

## 2. Materials and Methods

### 2.1. Experimental Setup

**Figure 1:**
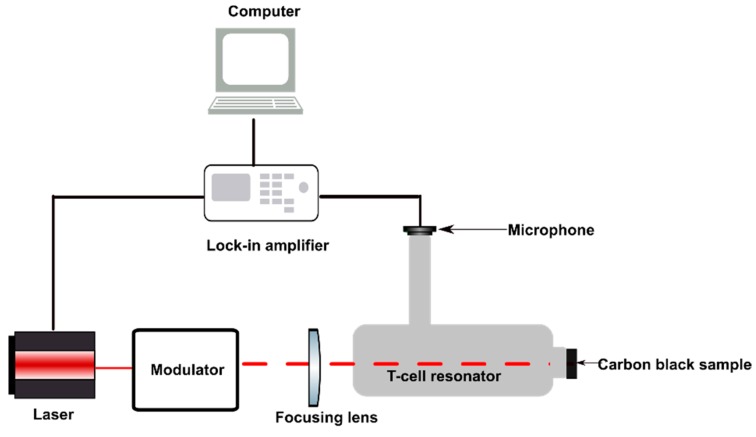
Schematic of the experimental setup.

The setup for the photoacoustic signal generation is represented in Figure 1. A diode laser (IMM photonics U-LD-651071A) served as the radiation source producing optical power of approximately 200 µW at a wavelength of 655 nm. A signal generator (Agilent technologies 33220A, Santa Clara, CA, USA) is used to modulate the laser with a 50% duty cycle. The beam is focused onto a carbon black probe using a spherical lens (f = 30 mm). Carbon black is a broad band absorber in the visible region and can therefore be excited with the selected laser. The generated PA signal is detected using a digital ultrasound MEMS microphone (Knowles SPH0641LU4H-1). The microphone output is a digital pulse density modulated (PDM) signal and is passed through a low-pass filter circuit for demodulation. The demodulated signal is fed into the lock-in-amplifier (Signal Recovery7265 DSP Lock-in-Amplifier) with a time constant of 100 ms to amplify and filter out noise from the generated PA signal. The modulation frequency is scanned between 8 kHz and 62 kHz at an increment of 10 Hz. For each frequency value, an average of 10 measurements were recorded with a computer. Two additional measurements were carried out between 8 kHz and 10 kHz. In one of those measurements the carbon black probe was replaced with a glass window and in the other measurement the laser was turned off to record the offset level.

#### 2.1.1. T-cell Resonator

**Figure 2:**
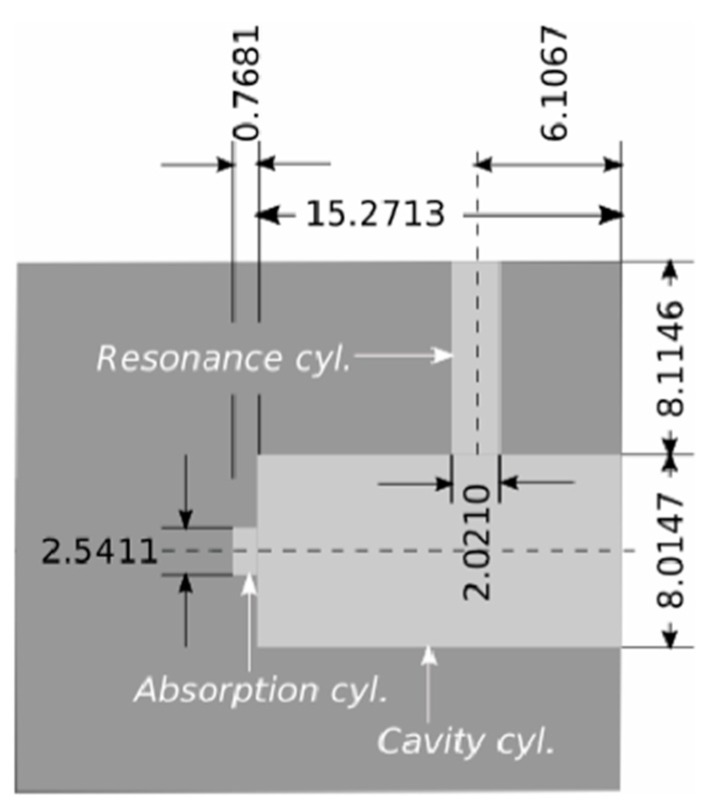
Cross-section of the T-cell resonator (light gray) showing the size of each cylinder in mm. The dimensions of the cells are the mean values obtained from a high precision measurement of the resonator [14].

The photoacoustic resonator consists of three interconnected cylinders as shown in Figure 2. A small absorption cylinder is longitudinally connected to a cavity cylinder, on which a resonance cylinder had been perpendicularly mounted, thus forming a T-like structure. The right end of the resonator where the laser beam enters is sealed using a polycarbonate window with an optical transmission of 85% in the visible region. The carbon black probe is pressed into the absorption cylinder, thus sealing the left end of the resonator. The upper end of the resonance cylinder is sealed by the microphone and its mounting.

**Figure 3:**
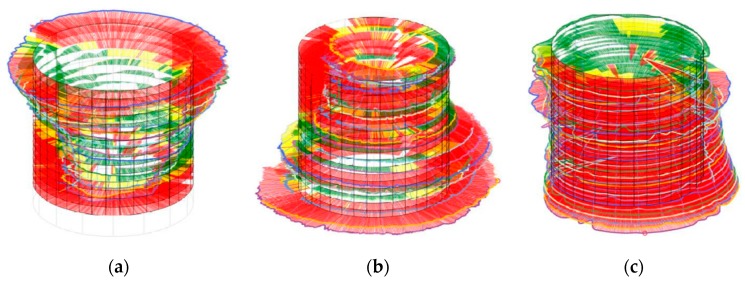
The unevenness of the diameter of the resonance cylinder, the absorption cylinder, and the cavity cylinder (left to right) resulting in asymmetrical cylinders [14]. The nominal values of the diameters are 2.0 mm, 2.5 mm and 8.0 mm respectively. Red/yellow/green: Deviation of the measured value and the nominal value larger than/similar to/smaller than 10 µm, respectively.

The resonator was manufactured by drilling out the cylinders from an aluminum block. Its dimensions were measured to check for geometrical imperfections using an optical scanner (ZEISS MICURA, ZEISS, Oberkochen, Germany) with a precision of less than 1 micrometer. Figure 3 shows that the diameter varied along each cylinder resulting in a rough interior surface. The deviations of the diameters were in the range of 0.01 mm to 0.06 mm and have been magnified by a factor of 2500 so that the unevenness of the cylinder is clearly visible.

#### 2.1.2. Microphone Operation

The PDM digital interface of the microphone used is designed to allow time multiplexing of two microphone outputs on a single data line using a single clock. This is achieved by transmitting the output pulse train of one microphone in the first half and of the other microphone in the second half of the sampling period. However, only one microphone is used in the setup. Therefore, no output signal is issued during one half of the sampling period, causing a discontinuous pulse train. For a low-pass filter used as a PDM digital to analog converter (DAC), the analog output voltage is the product of the DAC operating voltage and the PDM duty cycle. For the maximum microphone output signal, the pulse duty cycle is 50% because of the pulse discontinuity. Halving the maximum possible DAC output voltage lowers the DAC’s signal-to-noise ratio, which reduces the overall sensitivity of the setup. The digitization of the PA signal is done in two operation modes depending on the microphone’s clocking signal: the standard mode (detecting signals between 100 Hz and 10 kHz) and the ultrasonic mode (detecting signals between 10 kHz and 80 kHz). Switching between modes of operation requires changing the microphone’s clocking frequency. Therefore, continuous measurements between 8 kHz and 62 kHz are not possible and have to be divided in two parts.

Between 35 kHz to 60 kHz, the frequency response of the microphone is fairly flat with a difference of not more than 0.25 dB. Between 8 kHz to 35 kHz, the response had a resonance at around 26 kHz. 

### 2.2. Simulation Models

This section briefly describes how the two models are realized and implemented. Ideally, the simulation model would reflect the measured geometry (explained in the previous section) since the PA signal depends on the exact details of the resonator’s geometry. However, this would considerably increase the computation demands of the model and the simulation time. Therefore, an average diameter of each cylinder was calculated as seen in Figure 2 and used as the cylinder’s diameter during simulations. 

**Figure 4:**
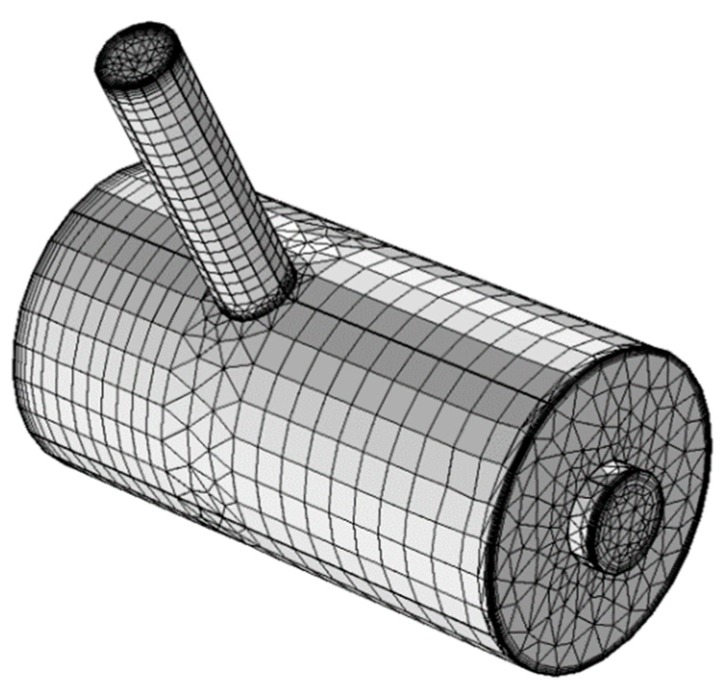
The resonator mesh (22,451 elements).

The design of the resonator is initially generated and meshed. We applied swept meshes in the resonance cylinder and in parts of the cavity cylinder, while a triangular mesh was used in areas of the resonator where a swept mesh could not be used. A structured mesh with swept meshing was chosen to reduce the number of mesh elements and enable faster computation. Even though the AME model does not require boundary layers, they were generated throughout the resonator since the same mesh was used for the VT model. A convergence study performed for the AME model indicated that the mesh depicted in Figure 4 is sufficient to obtain a good approximation to the actual solution.

#### 2.2.1. Amplitude Mode Expansion Model

The AME method is based on eigenmode expansion:

(1) $$p\left(r,\omega \right)=\underset{j}{\sum }A_{j}\left(\omega \right)p_{j}\left(r\right),$$

where p(r,ω) is the acoustic pressure at the measurement point r and the modulation frequency ω. It is executed using a MATLAB^®^ code which accesses COMSOL Multiphysics^®^ for calculations of the eigenmodes $p_{j}\left(r\right)$ and the eigenfrequencies $\omega_{j}$ of the resonator by solving the acoustic Helmholtz equation using a sound hard boundary condition. The amplitudes $A_{j}\left(\omega \right)$ are calculated using:

(2) $$A_{j}\left(\omega \right)=i\frac{A_{j}\omega }{\omega^{2}-\omega_{j}^{2}+i\omega \omega_{j}l_{j}},$$

where $A_{j}$ describes the excitation of the sound waves and is obtained by:

(3) $$A_{j}=\frac{\alpha \left(\gamma -1\right)}{V_{c}}\int_{V_{c}}p_{j}^{*}I\;dV,$$

where $V_{C}$ is volume of the resonator and I=I(r) is the laser intensity profile in the sample where the absorbing molecules are located. The variable γ is the ratio of isobaric and isochoric heat capacity and α is the absorption coefficient of the sample. The loss effects are introduced by loss factors $l_{j}$ in Equation (2), which account for the thermal and viscous losses at the bulk of the fluid and at the resonator’s surface, hindering $A_{j}\left(\omega \right)\to \infty$ as $\omega \to \omega_{j}$; in fact, due to the imaginary unit i, the amplitude always remains bounded. Detailed descriptions of the formulas can be found elsewhere [11,15]. Air is selected as the propagating fluid with the parameters in Table 1.

**Figure 5:**
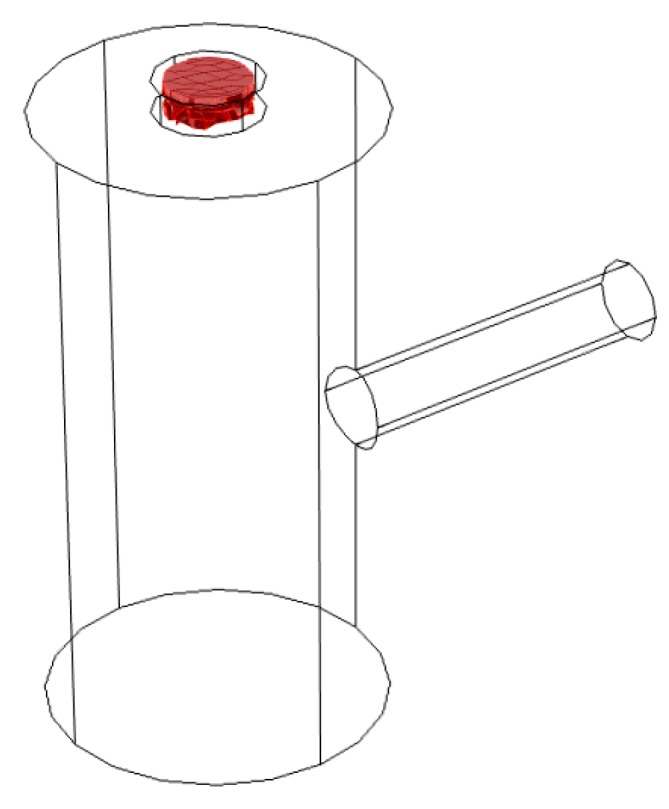
The position of the source term (red) within the resonator.

The PA signal was simulated between 8 kHz to 62 kHz with an increment of 10 Hz. The source term for sound generation is defined within the absorption cylinder as shown in Figure 5. Small variations of the shape and the size of the heat source have no significant effect on the simulation results [15].

#### 2.2.2. Viscothermal Model

The VT method is based on solving the Navier–Stokes equation, the continuity equation for the mass, and the energy balance equation. An equation of state is introduced to relate the variations in pressure, temperature and density. A detailed description of the equations can be found elsewhere [17]. The model is simulated using COMSOL Multiphysics^®^ software. 

The walls of the PA cell are set as sound hard (no slip and isothermal boundary conditions). Air was selected from the COMSOL Multiphysics^®^ material database as the propagating fluid in the resonator. The air properties like the speed of sound, dynamic viscosity, thermal conductivity, heat capacity at constant pressure and density are temperature dependent. The temperature was set to 20 °C and the static pressure to 1013 hPa. The source term was defined just like in the AME model.

The VT model had more variables than the AME model, hence it had 476,980 degrees of freedom while the AME model had 115,474. This is the reason why the VT model is considerably slower and computationally more demanding than the AME model. The PA signal of the VT model was calculated from 8 kHz to 62 kHz. Due to the long computing times the frequency increment was set to 50 Hz.

## 3. Results and Discussion

**Figure 6:**
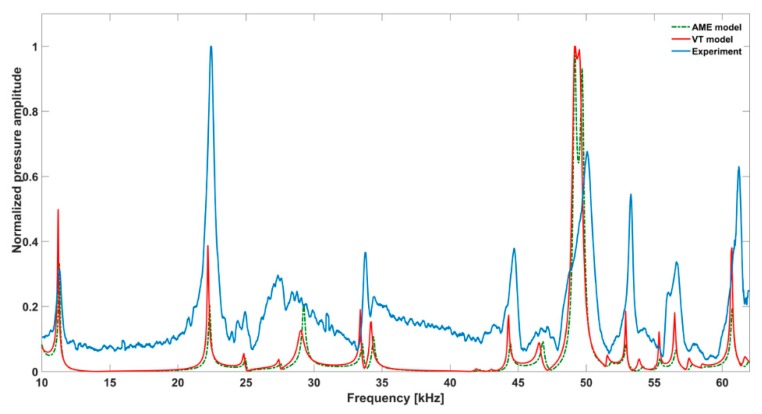
The frequency responses of the amplitude mode expansion (AME) model, the viscothermal (VT) model and the experimental measurement in the high frequency range.

**Figure 7:**
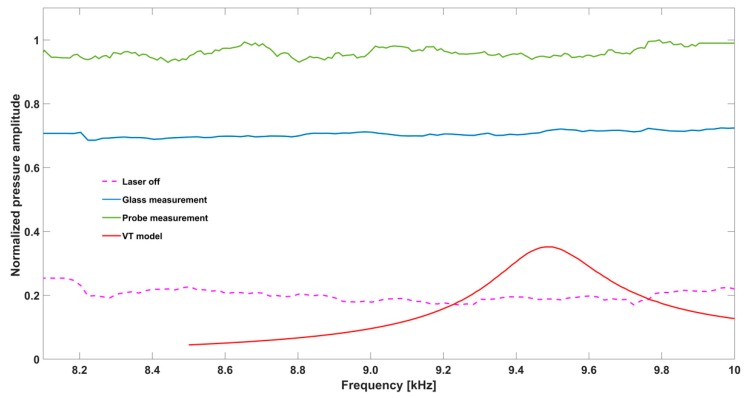
Experimental frequency response in the low frequency range. The pink plot is the offset level when the laser is turned off, the blue plot is the measurement with the glass window, the green plot is with the graphite probe while the red plot is the simulation from the viscothermal model.

As previously explained, the microphone has two operating modes and continuous measurements over the complete frequency range are not possible. The simulated and measured frequency response curves between 10 kHz to 62 kHz are shown in Figure 6. The experimental data was smoothed using the Savitzky Golay function in Matlab [18] and rescaled to enable easy comparison. The measurement results between 8 kHz to 10 kHz where, according to the simulations, the first resonance peak is expected are presented in Figure 7.

### 3.1. Measurement

The measurement is compared with the VT model since it is considered the more accurate simulation method. The resonance frequencies from the measurement show fairly good accordance with the VT simulations. The relative difference in the peak resonance frequency with respect to the value obtained from the VT model is not more than 1.1%. The temperature of the air inside the resonator was not tracked during the measurements. Temperature variations might be one of the reasons for the deviations since the speed of sound and hence the resonance frequencies depend on the actual temperature.

The widths of the measured resonances are larger than the widths obtained from the simulations. Table 2 compares the Q factors of the most prominent resonances. It indicates a considerably higher damping in the measurement system compared to the numerical model. The Q factor was estimated using:

(4) $$Q=\frac{f_{0}}{f_{h}-f_{l}},$$

where $f_{0}$ denotes the resonance frequency while $f_{l}$ and $f_{h}$ are the frequencies at which the value of the pressure amplitude has decreased to half of the resonance value. Due to the high damping, the measured Q values are obtained from the resonances where the Q factor could be estimated.

The damping can be attributed to leakage of the photoacoustic signal from the resonator. We suspect that the microphone mount does not seal the resonator tightly, and thus could be the location of the signal leakage. Furthermore, the resonator’s walls have a rough surface as indicated earlier, which increases the viscous losses at the surfaces. In cylindrical cells with a length to diameter ratio significantly larger than one, the surface roughness is important for longitudinal modes since the sound particle velocity is along the cylinder barrel [15]. The base noise level of the experimental response function is comparably high. This could have been caused by laser excitation of the resonator’s walls. The measurement system employs a diode laser, which characteristically has an elliptically divergent beam focused on the sample through a narrow absorption cylinder with a diameter of 2.5 mm. The edges of this beam will hit the walls of the resonator causing cell wall excitation. In addition, laser excitation of the PA cell’s window could be contributing to the noise level since the resonator does not have buffer zones.

Almost all the resonances that appeared in the response curves of the simulations are reproduced by the experimental measurement. The simulation model predicts two resonances between 30 kHz to 40 kHz. However, only a single resonance is measured in this region. The absence of one of the peaks can possibly be attributed to the fact that the resonance could not be distinguished from the base noise level, which is highest between 35 kHz to 40 kHz. The simulation model predicts two distinguishable resonances between 55 kHz to 57 kHz. However, broadening of the measured resonances has resulted in the merging of the two resonances. Unlike the simulation, the amplitude of the measured resonance at 22.2 kHz is the largest. However, the microphone response shows a resonance in this frequency range. Therefore, the strength of this measured resonance can be attributed to the synergetic amplification by the microphone and the resonator. 

Unlike the simulation, the measured response curve in Figure 7 does not clearly show a resonance between 8 and 10 kHz. The signal difference between the laser off measurement (red) to the glass measurement (green) supports our assumption that there is window and cell wall excitation.

### 3.2. Comparison of the Simulation Models

Both simulations were carried out with the same computer. The duration of a complete frequency sweep for the VT model (with 50 Hz increment) was 1 week while the AME model (with 10 Hz increments) took 7 min. All the VT model resonances are reproduced by the AME model with the peak frequency of the resonances having a relative difference of less than 1.8%. The broad resonance peak between 48 kHz andto 51 kHz was a result of two overlapping resonance peaks at 49.2 kHz to 49.5 kHz. At low modulation frequencies the resonances are attributed mainly to longitudinal modes of the cavity cylinder. At frequencies above 50 kHz, radial modes are also supported by the cavity cylinder thus accounting for the increase in the number of resonances (Table 3).

Since sensing applications require a strong PA signal, we restricted the following discussion to the 14 resonances with amplitudes that exceed a relative value of $10^{-6}$. It can be observed that the relative height of the resonance amplitudes for both models depends on the primary location of the mode within the resonator. If the mode is mainly located in the resonance cylinder where the surface area to volume ratio is large, the AME model underestimates the losses in comparison to the VT model, which results in larger amplitudes than the latter (the ratio of AME–VT model resonance amplitude was >1).

If the mode is mainly located in the cavity cylinder, where the surface area to volume ratio is small, the AME model slightly overestimates the losses in comparison to the VT model (resonance amplitude ratio <1). In some cases, the mode occupies the cavity cylinder as well as the resonance cylinder. Then the amplitude ratio can be smaller or larger than 1. The relationship between the surface area to volume ratio and loss leads to the conclusion that the AME model tends to overestimate bulk loss effects and underestimate the surface loss effects in comparison to the VT model. In the case of modes spanning both cylinders, the two effects mitigate each other.

## 4. Conclusions

We have shown the ability of the AME method to accurately simulate the PA signal in T-cell resonators. The method provides a much quicker and computationally less demanding alternative to the VT method for photoacoustic simulations in macroscopic resonators. The measured resonances and simulated resonances showed fairly good accordance. The width of the measured resonances indicates the need for additional loss mechanisms to be implemented in the modelling. The results of this investigation provide a good basis for the application of the AME method to an open T-cell resonator. Since the opening of the resonator deteriorates the signal, and hence the detection sensitivity, this work could represent a fast tool for the optimization of the resonator and for the enhancement of the detection sensitivity for non-invasive blood glucose measurement.

## Figures and Tables

**Table 1:** Air parameters at a temperature of 20 °C and a static pressure of 1013 hPa [16].

Density	$1.2044\;\mathrm{kg}/m^{3}$
Sound velocity	343.2 m/s
Viscosity	1.814 × 10^−5^ Pa s
Coefficient of heat conduction	2.58 × 10^−2^ W/m K
Specific heat capacity at constant volume	7.1816 × 10^2^ J/kg K
Specific heat capacity at constant pressure	1.0054 × 10^3^ J/kg K

**Table 2:** Resonance Q factors of measurement and simulation.

$f_{\mathrm{res}}\;\mathrm{in}\;\mathrm{kHz}$	$Q_{sim}$	$Q_{meas}$
11.2	80	19
22.2	123	34
44.3	211	55
52.9	353	124
60.7	304	80

**Table 3:** Resonance frequency, corresponding mode, location of strong antinodes, and amplitude ratio for the 14 strongest resonances. The location of the antinodes was determined by lifting the lower limit of the depicted data range appropriately.

$f_{\mathrm{res}}\;\mathrm{in}\;\mathrm{kHz}$	|p| Profile of Acoustic Mode	Location of Antinodes	$\frac{A^{\mathrm{AME}}\left(\omega_{\mathrm{res}}\right)}{A^{\mathrm{VT}}\left(\omega_{\mathrm{res}}\right)}$
9.500	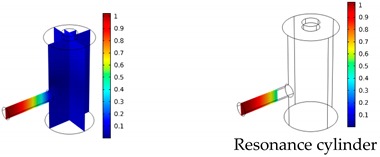		2.43
11.200	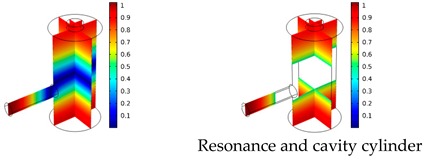		0.91
22.200	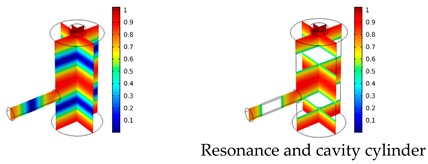		0.72
29.050	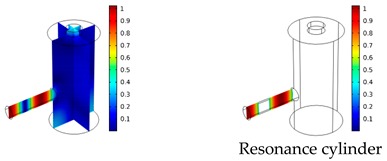		2.23
33.400	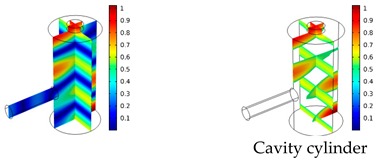		0.59
34.200	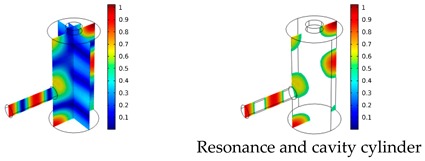		0.97
44.300	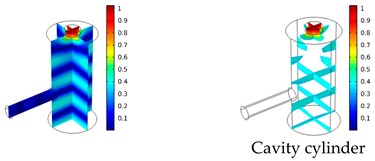		0.68
46.550	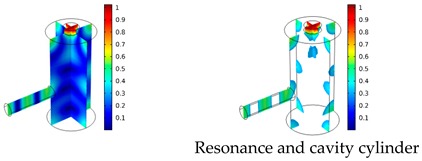		1.42
49.200	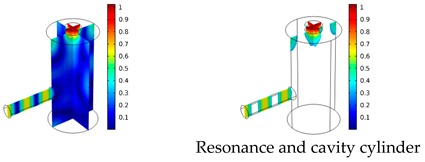		1.30
49.500	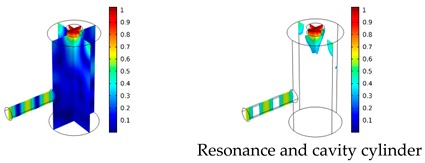		1.27
52.900	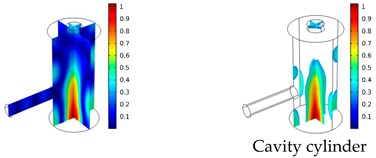		0.60
55.350	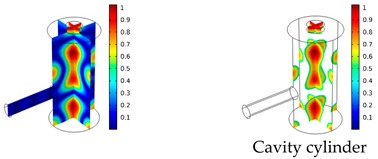		0.47
56.500	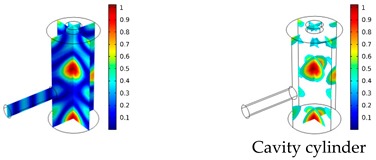		0.49
60.700	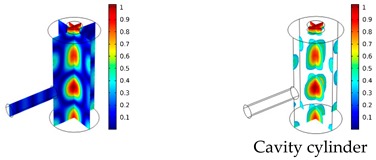		0.65
